# Comparison of Multimodal Therapies and Outcomes Among Patients With High-Risk Prostate Cancer With Adverse Clinicopathologic Features

**DOI:** 10.1001/jamanetworkopen.2021.15312

**Published:** 2021-07-01

**Authors:** Amar U. Kishan, R. Jeffrey Karnes, Tahmineh Romero, Jessica K. Wong, Giovanni Motterle, Jeffrey J. Tosoian, Bruce J. Trock, Eric A. Klein, Bradley J. Stish, Robert T. Dess, Daniel E. Spratt, Avinash Pilar, Chandana Reddy, Rebecca Levin-Epstein, Trude B. Wedde, Wolfgang A. Lilleby, Ryan Fiano, Gregory S. Merrick, Richard G. Stock, D. Jeffrey Demanes, Brian J. Moran, Michelle Braccioforte, Hartwig Huland, Phuoc T. Tran, Santiago Martin, Rafael Martínez-Monge, Daniel J. Krauss, Eyad I. Abu-Isa, Ridwan Alam, Zeyad Schwen, Albert J. Chang, Thomas M. Pisansky, Richard Choo, Daniel Y. Song, Stephen Greco, Curtiland Deville, Todd McNutt, Theodore L. DeWeese, Ashley E. Ross, Jay P. Ciezki, Paul C. Boutros, Nicholas G. Nickols, Prashant Bhat, David Shabsovich, Jesus E. Juarez, Natalie Chong, Patrick A. Kupelian, Anthony V. D’Amico, Matthew B. Rettig, Alejandro Berlin, Jonathan D. Tward, Brian J. Davis, Robert E. Reiter, Michael L. Steinberg, David Elashoff, Eric M. Horwitz, Rahul D. Tendulkar, Derya Tilki

**Affiliations:** 1 Department of Radiation Oncology, University of California, Los Angeles; 2 Department of Urology, University of California, Los Angeles; 3 Department of Urology, Mayo Clinic, Rochester, Minnesota; 4 Department of Radiation Oncology, Fox Chase Cancer Center, Philadelphia, Pennsylvania; 5 Department of Urology, University of Michigan, Ann Arbor; 6 Department of Urology, Brady Urological Institute, Johns Hopkins University, Baltimore, Maryland; 7 Department of Urology, Glickman Urological and Kidney Institute, Cleveland Clinic, Cleveland, Ohio; 8 Department of Radiation Oncology, Mayo Clinic, Rochester, Minnesota; 9 Department of Radiation Oncology, University of Michigan, Ann Arbor; 10 Department of Radiation Oncology, University of Toronto, Toronto, Canada; 11 Department of Radiation Oncology, Taussig Cancer Institute, Cleveland Clinic, Cleveland, Ohio; 12 Department of Oncology, Oslo University Hospital, The Norwegian Radium Hospital, Oslo, Norway; 13 Schiffler Cancer Center, Wheeling Hospital, Wheeling Jesuit University, Wheeling, West Virginia; 14 Radiation Oncology, Icahn School of Medicine at Mount Sinai, New York, New York; 15 Prostate Cancer Foundation of Chicago, Westmont, Illinois; 16 Martini-Klinik Prostate Cancer Center, University Hospital Hamburg Eppendorf, Hamburg, Germany; 17 Department of Radiation Oncology and Molecular Radiation Sciences, Johns Hopkins University School of Medicine, Baltimore, Maryland; 18 Department of Oncology, Clínica Universitaria de Navarra, University of Navarra, Pamplona, Spain; 19 William Beaumont School of Medicine, Oakland University, Royal Oak, Michigan; 20 Texas Oncology, Dallas; 21 Now with Department of Urology, Northwestern University Feinberg School of Medicine, Chicago, Illinois; 22 Department of Human Genetics, University of California, Los Angeles; 23 Department of Radiation Oncology, VA Greater Los Angeles Healthcare System, Los Angeles, California; 24 Department of Radiation Oncology, Dana-Farber Cancer Institute, Brigham and Women’s Hospital, Boston, Massachusetts; 25 Division of Hematology and Oncology, Department of Medicine, University of California, Los Angeles; 26 Department of Hematology and Oncology, VA Greater Los Angeles Healthcare System, Los Angeles, California; 27 Department of Radiation Oncology, Huntsman Cancer Institute, The University of Utah, Salt Lake City; 28 Department of Medicine Statistics Core, David Geffen School of Medicine, University of California, Los Angeles; 29 Department of Urology, University Hospital Hamburg-Eppendorf, Hamburg, Germany

## Abstract

**Importance:**

The optimal management strategy for high-risk prostate cancer and additional adverse clinicopathologic features remains unknown.

**Objective:**

To compare clinical outcomes among patients with high-risk prostate cancer after definitive treatment.

**Design, Setting, and Participants:**

This retrospective cohort study included patients with high-risk prostate cancer (as defined by the National Comprehensive Cancer Network [NCCN]) and at least 1 adverse clinicopathologic feature (defined as any primary Gleason pattern 5 on biopsy, clinical T3b-4 disease, ≥50% cores with biopsy results positive for prostate cancer, or NCCN ≥2 high-risk features) treated between 2000 and 2014 at 16 tertiary centers. Data were analyzed in November 2020.

**Exposures:**

Radical prostatectomy (RP), external beam radiotherapy (EBRT) with androgen deprivation therapy (ADT), or EBRT plus brachytherapy boost (BT) with ADT. Guideline-concordant multimodal treatment was defined as RP with appropriate use of multimodal therapy (optimal RP), EBRT with at least 2 years of ADT (optimal EBRT), or EBRT with BT with at least 1 year ADT (optimal EBRT with BT).

**Main Outcomes and Measures:**

The primary outcome was prostate cancer–specific mortality; distant metastasis was a secondary outcome. Differences were evaluated using inverse probability of treatment weight–adjusted Fine-Gray competing risk regression models.

**Results:**

A total of 6004 men (median [interquartile range] age, 66.4 [60.9-71.8] years) with high-risk prostate cancer were analyzed, including 3175 patients (52.9%) who underwent RP, 1830 patients (30.5%) who underwent EBRT alone, and 999 patients (16.6%) who underwent EBRT with BT. Compared with RP, treatment with EBRT with BT (subdistribution hazard ratio [sHR] 0.78, [95% CI, 0.63-0.97]; *P* = .03) or with EBRT alone (sHR, 0.70 [95% CI, 0.53-0.92]; *P* = .01) was associated with significantly improved prostate cancer–specific mortality; there was no difference in prostate cancer–specific mortality between EBRT with BT and EBRT alone (sHR, 0.89 [95% CI, 0.67-1.18]; *P* = .43). No significant differences in prostate cancer–specific mortality were found across treatment cohorts among 2940 patients who received guideline-concordant multimodality treatment (eg, optimal EBRT alone vs optimal RP: sHR, 0.76 [95% CI, 0.52-1.09]; *P* = .14). However, treatment with EBRT alone or EBRT with BT was consistently associated with lower rates of distant metastasis compared with treatment with RP (eg, EBRT vs RP: sHR, 0.50 [95% CI, 0.44-0.58]; *P* < .001).

**Conclusions and Relevance:**

These findings suggest that among patients with high-risk prostate cancer and additional unfavorable clinicopathologic features receiving guideline-concordant multimodal therapy, prostate cancer–specific mortality outcomes were equivalent among those treated with RP, EBRT, and EBRT with BT, although distant metastasis outcomes were more favorable among patients treated with EBRT and EBRT with BT. Optimal multimodality treatment is critical for improving outcomes in patients with high-risk prostate cancer.

## Key Points

**Question:**

Are external beam radiotherapy, with or without brachytherapy boost, or radical prostatectomy associated with differences in prostate cancer–specific mortality or distant metastasis outcomes in patients with high-risk prostate cancer after accounting for delivery of guideline-concordant multimodality therapy?

**Findings:**

In this cohort study of 6004 men with high-risk prostate cancer and at least 1 additional adverse clinicopathologic feature, no differences in prostate cancer-specific mortality across treatment modalities were identified among patients who received guideline-concordant multimodality therapy, although differences in time to metastasis were observed. However, significant differences in prostate cancer–specific mortality were identified when guideline-concordant multimodality care was not delivered.

**Meaning:**

These findings suggest that guideline-concordant multimodality care was associated with better prostate cancer–specific mortality outcomes for patients with high-risk prostate cancer.

## Teaser

This cohort study assesses prostate cancer–specific mortality and distant metastasis outcomes among men with high-risk prostate cancer treated with radical prostatectomy or external beam radiotherapy with or without brachytherapy boost.

## Introduction

High-risk localized prostate cancer, which is characterized by initial prostate-specific antigen (PSA) level greater than 20 ng/mL (to convert to micrograms per liter, multiply by 1), biopsy Gleason grade group 4 or 5 (formerly Gleason score 8-10) disease, and/or lesions at clinical T stage of T3 or greater,^1^ can be treated definitively with radiotherapy or radical prostatectomy (RP). Definitive radiotherapy options include external beam radiotherapy (EBRT) alone and EBRT with a brachytherapy boost (BT), both delivered with androgen deprivation therapy (ADT), while definitive RP may incorporate postoperative treatments. The optimal treatment remains controversial, with sparse prospective data^2,3^ and multiple retrospective series that included patients treated over multiple decades with strategies discordant with modern standard of care guidelines.^4,5,6^

Two recent studies focusing exclusively on patients with Gleason grade group 5 (formerly Gleason score 9-10) high-risk localized prostate cancer underscored the importance of enriching for modern treatment paradigms and highlighted the importance of multimodality therapy.^7,8^ However, while Gleason grade group 5 disease is uncommon, many patients with the considerably more heterogeneous entity of high-risk localized prostate cancer^9,10,11^ have added clinicopathologic factors that are associated with an added risk of treatment failure, including primary Gleason pattern 5 on biopsy, clinical T3b-4 disease, 50% of cores or more with biopsy results positive for prostate cancer, or at least 2 high-risk features. To comprehensively evaluate the associations between prostate cancer–specific mortality, distant metastasis, and the 3 standard treatments for high-risk prostate cancer while also accounting for clinical heterogeneity and delivery of guideline-concordant multimodality care, we established a large consortium of patients treated for high-risk localized prostate cancer with at least 1 adverse clinicopathologic feature across 16 tertiary centers from 2000 to 2014.

## Methods

This cohort study used deidentified data shared in concordance with the Health Insurance Portability and Accountability Act, with each institution’s institutional review board approving contribution of data to the coordinating data center (University of California, Los Angeles). The requirement for informed consent was waived by each institutional review board, given the retrospective nature of the study. This study followed the reporting requirements of the Strengthening the Reporting of Observational Studies in Epidemiology (STROBE) reporting guideline.

### Participants

Institutional and departmental databases from 16 tertiary referral centers were queried to identify patients with high-risk localized prostate cancer (as defined by National Comprehensive Cancer Network [NCCN] criteria, with T stage defined by physical examination) who received definitive treatment between 2000 and 2014. Patients with at least 1 adverse clinicopathologic feature were included in the analysis: primary Gleason pattern 5 disease on biopsy, at least 50% of cores positive for prostate cancer, cT3b-4, or at least 2 NCCN-defined high-risk features.^1^ We chose the NCCN risk stratification scheme since most participating institutions that maintained a database of patients with high-risk localized prostate cancer were based in the United States, where the NCCN stratification model is more commonly used.

### Exposure

Patients were broadly grouped into 3 cohorts based on the initial local treatment received: EBRT, EBRT with BT, or RP. These 3 cohorts were evaluated to identify subcohorts of patients who received guideline-concordant multimodality therapy by present-day standards. Patients treated with EBRT with at least 24 months of ADT were considered as having received optimal EBRT.^1^ Patients treated with EBRT with BT with at least 12 months of ADT were considered as having received optimal EBRT with BT.^1^ For patients receiving RP, optimal multimodality therapy depends on the presence of adverse pathological features, the timing of biochemical recurrence, and clinician preferences. Thus, the optimal RP group was defined as a collection of patients meeting the following criteria: biopsy Gleason grade group 5 disease and received adjuvant radiotherapy with ADT,^8^ pathologically identified lymph node–positive disease and received adjuvant ADT with or without adjuvant radiotherapy,^12^ experienced a biochemical recurrence and received salvage radiotherapy and/or salvage ADT, or never experienced a biochemical recurrence (considered to have been cured by RP alone).

### Outcomes

The primary outcome was prostate cancer–specific mortality, with distant metastasis as a secondary outcome. Prostate cancer–specific mortality was defined according to either clinical documentation or inclusion of prostate cancer as a primary cause of death on a death certificate. Imaging-based diagnosis of distant metastasis (typically performed on biochemical recurrence) was sufficient. Because comorbidity data were not widely available, all-cause mortality was not a main outcome of interest.^13^ Owing to the uncertain significance and variable definition of biochemical recurrence, it was not analyzed.

### Statistical Analysis

Differences in age and ADT duration across treatment cohorts were evaluated using a 2-tailed *t* test, whereas other continuous variables were compared using the Kruskal-Wallis test. The 2-tailed χ^2^ test was used to evaluate differences in categorical variables across cohorts. The reverse Kaplan-Meier method was used to calculate follow-up times.^14^ To adjust for potential ecological bias, continuous variables were centered by their mean values from each institutional cohort.^15^ Inverse probability of treatment weights (IPTWs) were calculated from a multinomial logistic regression with treatment cohorts as the outcome and including unfavorable (yes or no) and site-centered independent pretreatment prognostic covariates (ie, age at initial treatment, natural log of initial PSA, clinical T category, and Gleason grade group) as risk factors. In the entire cohort, 132 patients (2.2%) had missing information for at least 1 covariate (ie, age, cT stage, Gleason score, or initial PSA). Additionally, 300 patients (5.0%) of patients were missing data on prostate cancer–specific mortality status or time to prostate cancer–specific mortality information, and 120 patients (2.0%) were missing data on distant metastasis status or time to distant metastasis information. owing to the low amount of missing information, no imputation was performed for missing data. Weights were analyzed for extreme large values.

IPTW-adjusted Fine-Gray competing risk regression and Cox proportional hazard models were used to evaluate prostate cancer–specific mortality and distant metastasis outcome differences between treatment cohorts. Estimates are reported as subdistributional hazard ratios (sHRs) for Fine-Gray models or HRs for Cox models, along with their 95% CIs. All models were doubly robust.^16,17^ Patients were censored if they were lost to follow-up, if they had not experienced the event of interest at last follow-up, or if they experienced an event such that they were no longer at risk for the event of interest. The effect size of each independent covariate of interest across treatment groups was defined using the partial η^2^ metric and was calculated with and without applying IPTW-adjustment. Effect sizes of 0.14 or greater are considered large.^18^ All analyses were completed with SAS statistical software version 9.4 (SAS Institute). *P* values were 2-tailed, and statistical significance was set at *P* = .05. Data were analyzed in November 2020.

## Results

### Patient and Treatment Characteristics

A total of 6004 men with high-risk localized prostate cancer were analyzed, with a median (interquartile range [IQR]) age of 66.4 (60.9-71.8) years. There were 3175 patients who underwent RP (52.9%; median [IQR] age, 64.4 [58.5-69.1] years), 1830 patients who underwent EBRT alone (30.5%; median [IQR] age, 70 [63.7-75] years), and 999 patients who underwent EBRT with BT (16.6%; median [IQR] age, 67.7 [62.1-73] years). Full patient and treatment characteristics are presented in Table 1 and eTables 1, 2, and 3 in the Supplement. Among these, 1600 patients underwent optimal RP (50.4%), 879 patients underwent optimal EBRT alone (48.0%), and 461 patients underwent optimal EBRT with BT (46.1%). A total of 1395 patients who received EBRT alone (76.2%) and 897 patients who received EBRT with BT (89.7%) received ADT. The median (IQR) duration of ADT was significantly greater in patients receiving EBRT alone vs those receiving EBRT with BT (all patients: 22 [12-30] months vs 12 [4-24] months; *P* < .001; patients receiving optimal treatment: 28 [24-36] months vs 24 [18-24] months; *P* < .001). Among patients who received RP, 347 (10.9%) received adjuvant radiotherapy, while 847 patients (26.7%) received salvage radiotherapy; among patients receiving optimal RP, 198 patients (12.4%) received adjuvant radiotherapy and 511 patients (31.9%) received salvage radiotherapy.

**Table 1.** Patient Clinical Characteristics

Characteristic	No. (%)			*P* value^a^	No. (%)			*P* value^a^
	All RP (n = 3175)	All EBRT (n = 1830)	All EBRT + BT (n = 999)		Optimal RP (n = 1600)	Optimal EBRT (n = 879)	Optimal EBRT + BT (n = 461)	
Follow-up, median (IQR), y	4.24 (2.57-7.29)	8.17 (5.05-11.67)	8.70 (5.96-11.92)	<.001	4.59 (2.79-7.59)	8.85 (5.75-11.67)	9.31 (7.04-12.74)	<.001
Age, median (IQR), y	64.4 (58.5-69.1)	70.0 (63.7-75.6)	67.7 (62.1-72.7)	<.001	64.5 (58.6-68.8)	69.4 (63.1-75.2)	67.1 (61.2-72.1)	<.001
Initial PSA, median (IQR), ng/mL	11.2 (5.8-25.2)	15.0 (7.2-30.1)	18.6 (8.1-30.3)	<.001	12 (6.2-27.4)	15.2 (7.2-32.8)	22.3 (11.4-34.6)	<.001
Biopsy Gleason grade group								
1	124 (3.9)	63 (3.4)	74 (7.5)	<.001	104 (6.5)	32 (3.6)	47 (10.5)	<.001
2	229 (7.2)	215 (11.8)	89 (8.9)		185 (11.6)	99 (11.3)	47 (10.5)	
3	264 (8.3)	183 (10.0)	161 (16.3)		177 (11.1)	91 (10.4)	100 (22.3)	
4	1254 (39.6)	839 (45.9)	357 (36.2)		960 (60.3)	382 (43.5)	137 (30.5)	
5	1297 (40.9)	527 (28.8)	304 (30.9)		167 (10.5)	274 (31.2)	118 (26.3)	
Clinical tumor category								
1c	1407 (45.8)	459 (25.4)	210 (21.1)	<.001	736 (47.5)	177 (20.4)	85 (18.5)	<.001
2a	553 (17.4)	268 (14.8)	88 (8.8)		294 (18.4)	122 (13.9)	37 (8.1)	
2b	409 (13.3)	252 (13.9)	133 (13.4)		185 (11.9)	122 (13.9)	38 (8.3)	
2c	230 (7.5)	176 (9.7)	123 (12.3)		87 (5.6)	87 (9.9)	68 (14.8)	
3a	317 (10.3)	344 (18.8)	307 (30.8)		163 (10.5)	185 (21.3)	169 (36.8)	
3b	133 (4.3)	238 (13.2)	120 (12.0)		75 (4.8)	136 (15.7)	57 (12.4)	
4	21 (0.7)	70 (3.9)	15 (1.5)		9 (0.6)	40 (4.6)	5 (1.1)	

Abbreviations: BT, brachytherapy boost; EBRT, external beam radiotherapy; IQR, interquartile range; PSA, prostate-specific antigen; RP, radical prostatectomy.

SI conversion factor: To convert PSA to micrograms per liter, multiply by 1.

^a^ Continuous variables across treatments are compared using the Kruskal-Wallis test. The association between treatment and categorical variables are assessed using χ^2^ test of association.

Weighted and unweighted effect sizes for age, initial PSA, biopsy Gleason grade group, and clinical T stage among all patients and among patient who received optimal treatment are shown in eTable 4 in the Supplement. Prior to weighting, most variables had an effect size greater than 0.14. Patient characteristics after propensity score adjustment are shown in eTable 5 in the Supplement, and the distribution of propensity scores is shown in the eFigure in the Supplement. Crude, unadjusted, and adjusted cumulative incidence rate estimates for all patients are given in eTable 5 in the Supplement. Numbers at baseline differ for prostate cancer–specific survival and distant metastasis–free survival because not all patients had known cause of death information to compute prostate cancer–specific survival.

### Competing Risk Analyses

Crude, unadjusted, and cumulative incidence rate estimates are given in Table 2. Fine-Gray competing risks regression models are shown in Table 3, and IPTW-adjusted survival curves of time until prostate cancer–specific mortality and distant metastasis are shown in the Figure. Adjusted 5-year prostate cancer–specific mortality incidence rates were 5.3% (95% CI, 3.9%-7.2%) for all patients who received RP, 4.6% (95% CI, 3.7%-5.8%) for all patients who received EBRT alone, and 4.0% (95% CI, 3.0%-5.4%) for all patients who received EBRT with BT. Compared with patients who received RP, those who received EBRT with BT had reduced risk of prostate cancer–specific mortality (sHR, 0.70 [95% CI, 0.53-0.92]; *P* = .01), as did those who received EBRT alone (sHR, 0.78 [95% CI, 0.63-0.97]; *P* = .03). No significant differences in prostate cancer–specific mortality were found between patients treated with EBRT with BT vs EBRT alone (sHR, 0.89 [95% CI, 0.67-1.18]; *P* = .43). Adjusted 5-year incidence rates of distant metastasis were 18.4% (95% CI, 16.7%-20.2%) for patients who received RP, 11.7% (95% CI, 10.1%-13.5%) for patients who received EBRT alone, and 6.8% (95% CI, 5.4%-8.4%) for patients who received EBRT with BT. Compared with patients who received RP, risk of distant metastasis was reduced among patients who received EBRT alone (sHR, 0.50 [95% CI, 0.44-0.58]; *P* < .001) and those who received EBRT with BT (sHR, 0.25 [95% CI, 0.16-0.37]; *P* < .001). Compared with patients who received EBRT alone, those who received EBRT with BT also had reduced risk of distant metastasis (sHR, 0.52 [95% CI, 0.33-0.80]; *P* = .003).

**Table 2.** Crude Event Rates and Cumulative and Adjusted Cumulative Incidence Estimate Rates

Treatment cohort	Evaluable patients at time 0^a^	Time interval, y	Events, No.	Competing risk events, No.	Patients censored, No.^b^	No. at risk (end of interval)	Cumulative incidence at end of interval (95% CI)^c^	Adjusted cumulative incidence at end of interval (95% CI)^d^
**Prostate cancer-specific mortality**								
All RP	2892	0-5	99	194	1561	1038	5.2 (4.3-6.2)	5.3 (3.9-7.2)
		5-10	55	77	666	240	13.3 (11.7-15.2)	13.3 (10.7-16.7)
All EBRT	1767	0-5	67	151	403	1146	4.0 (3.2-5.0)	4.6 (3.7-5.8)
		5-10	66	136	489	455	10.3 (8.9-12.1)	11.7 (9.7-14.2)
All EBRT+BT	911	0-5	24	62	184	641	3.6 (2.8-4.6)	4.1 (3.6-5.4)
		5-10	34	74	318	215	9.3 (7.4-11.7)	10.3 (8.1-13.1)
Optimal RP	1462	0-5	35	101	764	562	3.6 (2.8-4.6)	3.4 (2.4-4.8)
		5-10	18	45	365	134	9.3 (7.3-11.9)	8.2 (6.3-10.6)
Optimal EBRT	852	0-5	28	53	166	605	3.3 (2.4-4.5)	3.3 (2.3-4.8)
		5-10	31	63	260	251	8.4 (6.5-11.3)	8.1 (6.0-10.7)
Optimal EBRT+BT	402	0-5	7	19	58	318	2.8 (2.1-3.9)	3.4 (2.3-5.0)
		5-10	13	37	153	115	7.4 (5.3-10.3)	8.1 (5.6-11.7)
**Distant metastasis**								
All RP	2929	0-5	452	197	1352	928	20.3 (18.8-22.1)	18.4 (16.7-20.2)
		5-10	109	72	540	207	32.7 (30.3-35.3)	30.2 (27.6-33.1)
All EBRT	1793	0-5	176	152	384	1081	11.2 (9.8-12.3)	11.7 (10.1-13.5)
		5-10	81	143	435	422	18.4 (16.5-20.4)	19.7 (17.3-22.4)
All EBRT+BT	973	0-5	61	74	172	666	6.3 (5.2-7.6)	6.8 (5.4-8.4)
		5-10	26	106	302	232	10.7 (8.8-13.0)	11.6 (9.5-14.3)
Optimal RP	1539	0-5	163	102	750	524	15 (13.3-17.12)	13.4 (11.7-15.4)
		5-10	56	45	309	114	26.8 (23.7-30.2)	23.5 (20.3-27.2)
Optimal EBRT	863	0-5	76	53	158	576	9.1 (7.5-11.1)	8.7 (7.1-10.6)
		5-10	42	66	234	234	16.7 (14.3-19.6)	16.1 (13.4-19.3)
Optimal EBRT+BT	447	0-5	18	27	55	347	3.9 (2.5-5.9)	4.2 (2.8-6.2)
		5-10	12	57	149	129	7.3 (5.3-10.1)	7.8 (5.5-11.1)

Abbreviations: BT, brachytherapy boost; EBRT, external beam radiotherapy; RP, radical prostatectomy.

^a^ Numbers at baseline differ for prostate cancer-specific survival and distant metastasis-free survival because not all patients had known cause-of-death information to compute prostate cancer–specific survival.

^b^ Patients were censored if they were lost to follow-up, if they had not experienced the event of interest at last follow-up, or if they experienced an event such that they were no longer at risk for the event of interest.

^c^ Unadjusted cumulative incidence estimates were generated Fine and Gray methods for competing risk.

^d^ Adjusted cumulative incidence estimates were generated using Fine and Gray methods for competing risk with inverse probability of treatment weights, calculated using propensity scores that were determined using multinomial logistic regression with treatment cohort as the outcome and site-centered age at treatment, natural log of initial prostate-specific antigen level, clinical T stage, and Gleason grade group as pretreatment, prognostic covariates.

**Table 3.** Competing Risk Models of Time Until Prostate Cancer–Specific Mortality and Distant Metastasis

Comparison	Subdistribution hazard ratio (95% CI)^a^	*P* value
**All patients**		
Prostate cancer specific mortality		
All EBRT vs all RP	0.78 (0.63-0.97)	.03
All EBRT+BT vs all RP	0.70 (0.53-0.92)	.01
All EBRT+BT vs all EBRT	0.89 (0.67-1.18)	.43
Distant metastasis		
All EBRT vs all RP	0.50 (0.44-0.58)	<.001
All EBRT+BT vs all RP	0.29 (0.23-0.36)	<.001
All EBRT+BT vs all EBRT	0.58 (0.46-0.73)	<.001
**Optimal treatment**		
Prostate cancer specific mortality		
Optimal EBRT vs optimal RP	0.76 (0.52-1.09)	.14
Optimal EBRT+BT vs optimal RP	0.86 (0.55-1.34)	.50
Optimal EBRT+BT vs optimal EBRT	1.13 (0.69-1.85)	.62
Distant metastasis		
Optimal EBRT vs optimal RP	0.48 (0.38-0.61)	<.001
Optimal EBRT+BT vs optimal RP	0.25 (0.16-0.37)	<.001
Optimal EBRT+BT vs optimal EBRT	0.52 (0.33-0.80)	.003

Abbreviations: BT, brachytherapy boost; EBRT, external beam radiotherapy; RP, radical prostatectomy.

^a^ Models are adjusted with inverse probability of treatment weights. The factors in the model include treatment and age at treatment, natural log of initial prostate-specific antigen level, clinical T stage, and Gleason grade group.

**Figure.**
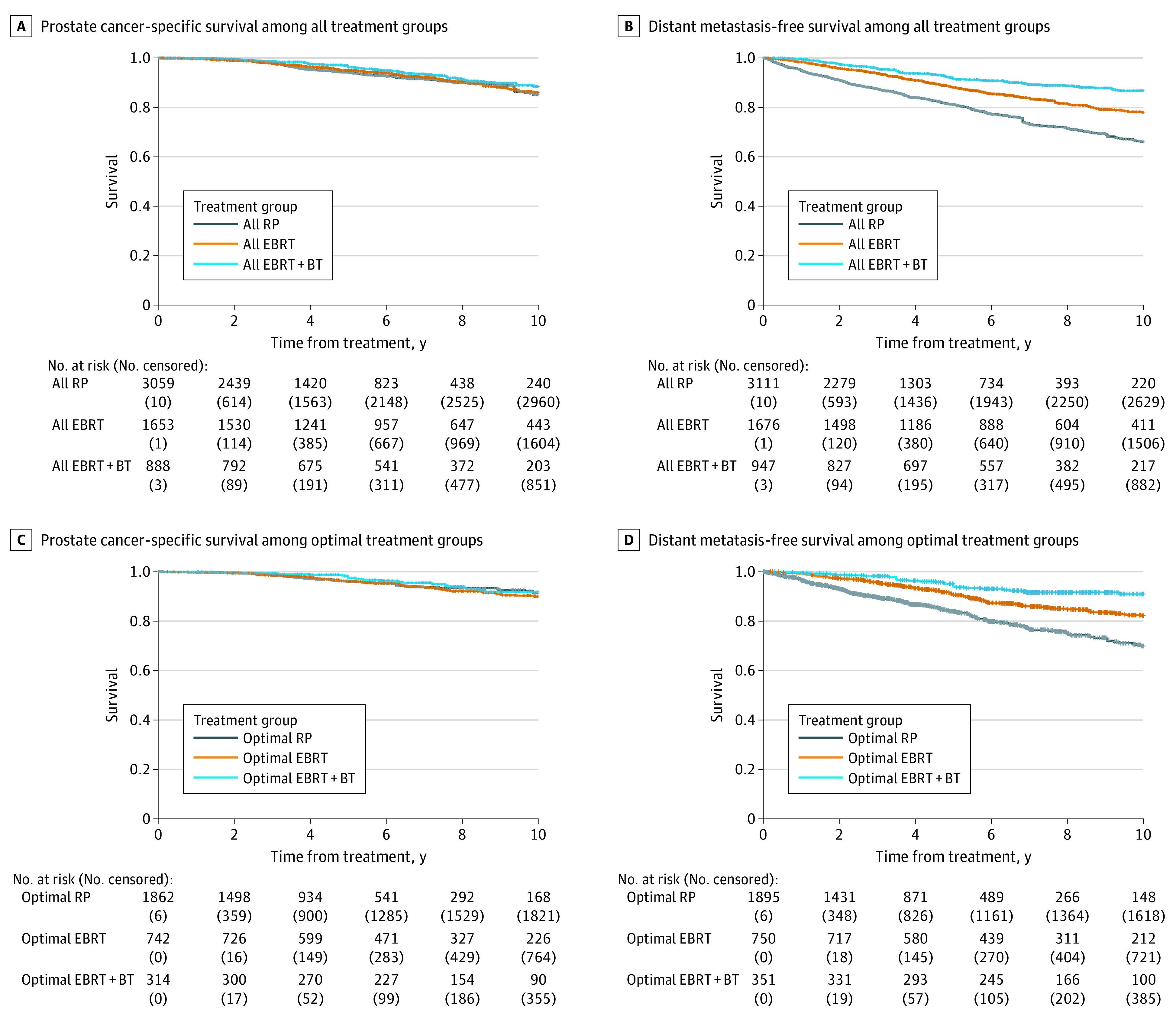
Adjusted Survival Curves for Prostate Cancer–Specific Survival and Distant Metastasis–Free Survival, Weighted by the Inverse Probability of Treatment Adjusted curves were generated with reverse Kaplan-Meier methods with IPTW, calculated with propensity scores that were determined by using multinomial logistic regression with treatment cohort as the outcome and site-centered age at treatment, natural log of initial prostate-specific antigen, clinical T stage, and Gleason Grade group as pretreatment prognostic covariates. BT indicates brachytherapy boost; EBRT, external beam radiotherapy; and RP, radical prostatectomy.

We repeated these analyses in the subcohorts of patients who underwent optimal therapy as defined by guideline-concordant care by modern standards. Fine-Gray competing risks regression models are shown in Table 3. Adjusted 5-year prostate cancer–specific mortality incidence rates were 3.4% (95% CI, 2.4%-4.8%) among patients who received optimal RP, 3.3% (95% CI, 2.3%-4.8%) among patients who received optimal EBRT alone, and 3.4% (95% CI, 2.3%-4.8%) among patients who received optimal EBRT with BT. No significant differences in prostate cancer–specific mortality were found between any of the cohorts (eg, optimal EBRT vs optimal RP: sHR, 0.76 [95% CI, 0.52-1.09]; *P* = .14) (Table 3). Adjusted 5-year incidence rates of distant metastasis were 13.4% (95% CI, 11.7%-15.4%) for optimal RP, 8.7% (95% CI, 7.1%-10.6%) for optimal EBRT alone, and 4.2% (95% CI, 2.8%-6.2%) for optimal EBRT with BT. Compared with patients who received optimal RP, there was reduced hazard for time to distant metastasis among patients who received optimal EBRT alone (sHR, 0.48 [95% CI, 0.38-0.61]; *P* < .001) or optimal EBRT with BT (sHR, 0.25 [95% CI, 0.16-0.37]; *P* < .001). Additionally, compared with those who received optimal EBRT alone, those who received optimal EBRT with BT had reduced risk of distant metastasis (sHR, 0.52 [95% CI, 0.33-0.80]; *P* = .003). Cause-specific analyses and all-cause mortality analyses are presented in eTable 5 and eTable 6 in the Supplement.

### Sensitivity Analysis Focused on Long-term Follow-up

Given the significant differences in follow-up and potential differences in distant metastasis and prostate cancer–specific mortality development that might occur over time, we repeated all analyses on a subgroup of 3273 patients with high-risk localized prostate cancer who had a minimum of 4.5 years of follow-up. Fine-Gray competing risks regression models for all patients and patients who received NCCN-defined optimal treatment are shown in Table 4. The results were essentially unchanged from overall analyses.

**Table 4.** Competing Risk Models of Time Until Prostate Cancer–Specific Mortality and Distant Metastasis Among Patients With Greater Than 4.5 Years of Follow-up

Comparison	Subdistribution hazard ratio (95% CI)^a^	*P* value
**All patients (n = 3187)**		
Prostate cancer specific mortality		
All EBRT vs all RP	0.74 (0.55-0.99)	.045
All EBRT+BT vs all RP	0.75 (0.52-1.07)	.12
All EBRT+BT vs all EBRT	1.02 (0.71-1.45)	.92
Distant metastasis		
All EBRT vs all RP	0.53 (0.44-0.63)	<.001
All EBRT+BT vs all RP	0.28 (0.21-0.36)	<.001
All EBRT+BT vs all EBRT	0.52 (0.39-0.69)	<.001
**Optimal treatment (n = 1673)**		
Prostate cancer specific mortality		
Optimal EBRT vs optimal RP	0.63 (0.38-1.04)	.07
Optimal EBRT+BT vs optimal RP	0.99 (0.58-1.69)	.97
Optimal EBRT+BT vs optimal EBRT	1.56 (0.87-2.79)	.13
Distant metastasis		
Optimal EBRT vs optimal RP	0.47 (0.35-0.63)	<.001
Optimal EBRT+BT vs optimal RP	0.28 (0.18-0.44)	<.001
Optimal EBRT+BT vs optimal EBRT	0.59 (0.36-0.95)	.03

Abbreviations: BT, brachytherapy boost; EBRT, external beam radiotherapy; RP, radical prostatectomy.

^a^ Models are adjusted with inverse probability of treatment weights. The factors in the model include treatment and age at treatment, natural log of initial prostate specific antigen level, clinical T stage, and Gleason Grade group.

## Discussion

In this cohort study of patients with high-risk localized prostate cancer who had at least 1 adverse clinicopathologic feature, no significant differences in prostate cancer–specific mortality were identified in patients with who received optimal, modern guideline-concordant treatment, regardless of whether the primary therapy was EBRT with BT, EBRT alone, or RP. However, reflective of evolving standards of care over time, only approximately half of patients received this type of care; when all patients were included, EBRT with BT and EBRT alone were associated with improved prostate cancer–specific mortality compared with RP. Both radiotherapeutic modalities were associated with longer time to distant metastasis than RP, regardless of delivery of guideline-concordant multimodality care. When restricted to patients with longer follow-up, the results persisted.

The relatively consistent difference in distant metastasis outcomes between RP-based management and radiotherapy-based management, which largely included at least 12 months of ADT by definition, suggests that treatment in patients receiving RP can be further optimized, potentially with more frequent incorporation of systemic therapy. Notably, there may be an interval of 5 to 6 years from the onset of distant metastasis to a prostate cancer–specific mortality event; therefore, with longer follow-up, differences in distant metastasis may translate to differences in prostate cancer–specific mortality.^19,20^ However, this phenomenon was not seen in our sensitivity analysis that enriched for longer follow-up. Overall, the persistent difference in distant metastasis outcomes, even after accounting for optimal therapy, mirrors prior findings restricted to Gleason grade group 5 disease, particularly when analysis was restricted to patients receiving optimal treatment in the EBRT cohort.^7^ There are several possible explanations, which are not mutually exclusive. First, this might indicate a true benefit in systemic control, mediated by improved local control^21^ or an immunomodulatory impact of radiation that might be most pronounced with BT.^22^ Second, distant metastasis events might have been detected more frequently in patients who received RP owing to a lower threshold for biochemical recurrence and thus for imaging. Third, it might reflect a lead-time afforded by ADT. However, the median duration of follow-up was significantly longer with either EBRT or EBRT with BT than with RP by margins that greatly exceeded the median duration of ADT.

Importantly, most comparative outcomes data for high-risk localized prostate cancer are from retrospective studies that have reported conflicting results with respect to prostate cancer–specific mortality outcomes and did not account for modern standard of care delivery.^4,5,6^ The only 2 published prospective studies comparing definitive modalities, to our knowledge, include a randomized clinical trial by Lennernäs et al^2^ of 89 patients receiving of RP or EBRT with BT and a nonrandomized comparison by Neal et al^3^ of 414 patients with locally advanced cancers who were excluded from the Protect study but were followed-up after EBRT or RP. Neither study found significant prostate cancer–specific mortality differences between modalities, but the study by Lennernäs et al^2^ was underpowered and included 6 months of ADT in both arms, while the study by Neal et al^3^ did not report on specifics of multimodality care and did not specifically evaluate patients with high-risk localized prostate cancer. Two recent retrospective studies focused on the specific subgroup of patients with Gleason grade group 5 disease.^7,8^ In a 2018 study of 1809 men with Gleason grade group 5 disease, Kishan et al^7^ found improved prostate cancer–specific mortality and distant metastasis outcomes in patients treated with EBRT with BT compared with those treated with EBRT alone or RP. A 2019 study by Tilki et al^8^ compared outcomes in an independent population of 639 men with Gleason grade group 5 disease treated with EBRT with BT or RP. Overall, Tilki et al^8^ found that EBRT with BT was associated with improved prostate cancer–specific mortality outcomes compared with RP, but the small cohort of 50 patients who received RP followed by adjuvant radiotherapy and ADT had comparable outcomes with the patients receiving EBRT with BT. Unlike these previous reports, this study included patients with a standard consensus-based diagnosis of high-risk localized prostate cancer and at least 1 adverse clinicopathologic feature, and included consideration of multimodality treatment paradigms.

The clear differences from accounting for guideline-concordant multimodality treatment in conclusions regarding prostate cancer–specific mortality outcomes is a novel finding. Prior analyses have not addressed this owing to critical limitations regarding the quality of initial treatment data, particularly related to the use and duration of ADT, as well as unclear definitions of optimal therapy in patients undergoing RP. This study supports the idea that modern, guideline-concordant multimodality care is critical to obtain optimal outcomes for men with high-risk localized prostate cancer. A key feature of our definition of optimal RP is in fact the inclusion of patients who did not experience a recurrence and were thus correctly spared postoperative therapy and its attendant toxic effects.^23^

### Limitations

This study has several limitations. First, the data were gathered retrospectively, and attempts to minimize bias using statistical techniques, such as propensity score adjustments and site-centered covariates, are unable to eliminate the significant selection biases, detection biases, and ecological biases that will impact any multi-institutional retrospective study comparing distinct treatment options. There is very likely to have been ascertainment bias in the diagnosis of distant metastasis across centers, given the nonuniform follow-up and potential differential use of advanced nuclear imaging scans to identify distant metastasis. Since not all centers included in this analysis treated patients with all 3 modalities, ecological biases may be significant. Residual differences even after propensity score adjustment suggest that confounding by indication was still substantial. Thus, prospective data are needed to validate the findings.

Second, while our definitions of optimal therapy were evidence-based, there may not be universal agreement on how these definitions were reached. For optimal RP, we chose to include patients who did not have a recurrence after RP, as these patients may have been cured by RP alone and likely did not need treatment intensification. Excluding this subgroup of patients from the optimal RP would lead to increased distant metastasis and prostate cancer–specific mortality outcomes in that cohort, biasing against the optimal RP group. Limitations related to lack of presalvage radiotherapy PSA and use of ADT with salvage radiotherapy might have affected our ability to evaluate distant metastasis outcomes.^24,25^ For optimal EBRT, a minimum of 18 months of ADT (vs 24 months) has been supported by the Prostate Cancer Study IV randomized trial,^26^ but this had not yet been accepted as a standard of care option when our analyses were designed. For optimal EBRT with BT, no randomized clinical trial has evaluated the duration of ADT, to our knowledge; therefore, we used the minimum duration recommended by the NCCN for classification as optimal EBRT with BT. Longer durations may be appropriate.^27^ Because the definition of optimal EBRT and EBRT with BT required minimum durations of ADT, an immortality bias in favor of these groups was introduced. However, the persistence of our results in the sensitivity analysis, as well as the overall longer follow-up in the EBRT and EBRT with BT groups compared with the RP group argues against such a bias influencing our results. The inclusion of patients receiving salvage radiotherapy in the optimal RP group also may have introduced some bias against this group, as such patients by definition had concern for a biochemical recurrence. However, the use of salvage radiotherapy is considered a key part of multimodality RP-based therapy, and omitting such patients might have artificially enriched for patients cured by RP alone. Additionally, clinical stage can have high interobserver variability and is difficult to standardize.^28^

## Conclusions

This cohort study found that among patients with high-risk prostate cancer receiving guideline-concordant multimodal therapy, prostate cancer–specific mortality outcomes were equivalent among RP, EBRT alone, and EBRT with BT. Strategies to minimize distant metastasis are warranted, particularly in patients pursuing RP.

## Notes

Supplement. **eFigure.** Distribution of Propensity Scores **eTable 1.** Patient Contribution and Median Follow-up by Overall Treatment Cohort, Stratified by Institution **eTable 2.** Pathological Characteristics and Treatment Details **eTable 3.** Specific Breakdown of Presence of Adverse Clinicopathologic Features by Cohort **eTable 4.** Covariate Effect Sizes Before and After Inverse Probability of Treatment Weighting Across Treatment Groups **eTable 5.** Clinical Characteristics After Propensity Score Adjustment **eTable 6.** Cause-Specific Regression Models of Time Until Prostate Cancer-Specific Mortality and Distant Metastasis **eTable 7.** Cause-Specific Regression Models of Time Until All-Cause Mortality
