# Supplementary material for: Comparison of Multimodal Therapies and Outcomes Among Patients With High-Risk Prostate Cancer With Adverse Clinicopathologic Features
Source: JAMA Netw Open. 2021 Jul 1;4(7):e2115312. doi: 10.1001/jamanetworkopen.2021.15312 (PMC8251338; doi:10.1001/jamanetworkopen.2021.15312)
Supplement: Supplement. — eFigure. Distribution of Propensity Scores eTable 1. Patient Contribution and Median Follow-up by Overall Treatment Cohort, Stratified by Institution eTable 2. Pathological Characteristics and Treatment Details eTable 3. Specific Breakdown of Presence of Adverse Clinicopathologic Features by Cohort eTable 4. Covariate Effect Sizes Before and After Inverse Probability of Treatment Weighting Across Treatment Groups eTable 5. Clinical Characteristics After Propensity Score Adjustment eTable 6. Cause-Specific Regression Models of Time Until Prostate Cancer-Specific Mortality and Distant Metastasis eTable 7. Cause-Specific Regression Models of Time Until All-Cause Mortality [file jamanetwopen-e2115312-s001.pdf]

Supplemental Online Content

Kishan AU, Karnes RJ, Romero T, et al. Comparison of multimodal therapies and outcomes among patients with high-risk prostate cancer with adverse clinicopathologic features. *JAMA Netw Open*. 2021;4(7):e2115312. doi:10.1001/jamanetworkopen.2021.15312

eFigure. Distribution of Propensity Scores

eTable 1. Patient Contribution and Median Follow-up by Overall Treatment Cohort, Stratified by Institution

eTable 2. Pathological Characteristics and Treatment Details

eTable 3. Specific Breakdown of Presence of Adverse Clinicopathologic Features by Cohort

eTable 4. Covariate Effect Sizes Before and After Inverse Probability of Treatment Weighting Across Treatment Groups

eTable 5. Clinical Characteristics After Propensity Score Adjustment

eTable 6. Cause-Specific Regression Models of Time Until Prostate Cancer-Specific Mortality and Distant Metastasis

eTable 7. Cause-Specific Regression Models of Time Until All-Cause Mortality

This supplemental material has been provided by the authors to give readers additional information about their work.

© 2021 Kishan AU et al. *JAMA Network Open.*

eFigure. Distribution of Propensity Scores

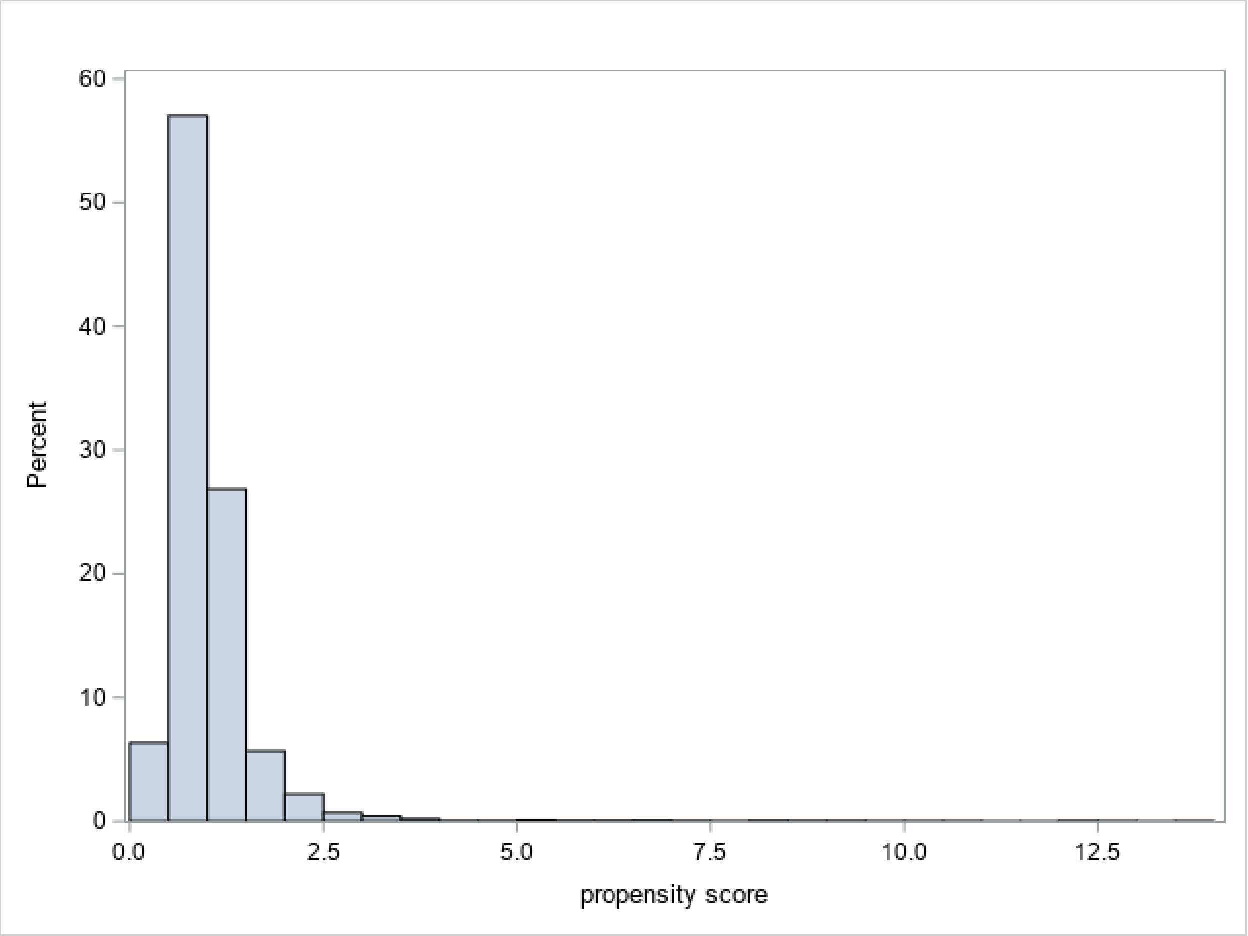

The mean propensiy score was 0.88 (standard deviation, 0.7), with a median score of 0.88. The range of scores were 0.23-13.68, with an interquartile range of 0.7-1.15.

© 2021 Kishan AU et al. *JAMA Network Open.*

eTable 1. Patient Contribution and Median Follow-up by Overall Treatment Cohort, Stratified by Institution

Institution	Total 6004	RP (n=3175)	EBRT (n=1830)	EBRT+BT (n=999)	Median follow-up time (years) (interquartile range)
University of California, Los Angles	293 (5 %)	153 (5 %)	117 (6 %)	23 (2 %)	7.3 (5.6 - 11.8)
California Endocurie Therapy Center	194 (3 %)	0 (0 %)	0 (0 %)	194 (19 %)	9.2 (5.8 - 13)
Fox Chase Cancer Center	651 (11 %)	152 (5 %)	495 (27 %)	4 (0 %)	5.5 (3.3 - 8.2)
Icahn School of Medicine at Mount Sinai	146 (2 %)	0 (0 %)	14 (1 %)	132 (13 %)	7.9 (4.4 - 10.5)
Cleveland Clinic	513 (9 %)	257 (8 %)	253 (14 %)	3 (0 %)	6.1 (2.8 - 10.2)
Wheeling Jesuit University	58 (1 %)	0 (0 %)	0 (0 %)	58 (6 %)	8.4 (5.4 - 13.5)
University of Michigan	387 (6 %)	0 (0 %)	352 (19 %)	35 (4 %)	8.5 (6 - 12.1)
Johns Hopkins University School of Medicine	260 (4 %)	184 (6 %)	76 (4 %)	0 (0 %)	10.6 (9 - 12.1)
Oslo University Hospital	234 (4 %)	0 (0 %)	69 (4 %)	165 (17 %)	8 (7 - 9.7)
William Beaumont Hospital	84 (1 %)	0 (0 %)	0 (0 %)	84 (8 %)	6.2 (4 - 8.3)
Chicago Prostate Institute	81 (1 %)	0 (0 %)	0 (0 %)	81 (8 %)	8.3 (6 - 13.7)
CUN Navarra	155 (3 %)	0 (0 %)	0 (0 %)	155 (16 %)	13.8 (11.2 - 15.5)
Mayo Clinic	659 (11 %)	594 (19 %)	50 (3 %)	15 (2 %)	6 (3.9 - 11.6)
Hamburg	1740 (29 %)	1740 (55 %)	0 (0 %)	0 (0 %)	3.1 (2 - 5)
Princess Margaret Hospital	346 (6 %)	0 (0 %)	342 (19 %)	4 (0 %)	10.9 (7.8 - 12.9)
University of Utah	203 (3 %)	95 (3 %)	62 (3 %)	46 (5 %)	8.9 (5.6 - 12.3)

EBRT, external beam radiotherapy; EBRT+BT, external beam radiotherapy with brachytherapy boost; n, number; RP, radical prostatectomy

© 2021 Kishan AU et al. *JAMA Network Open.*

eTable 2. Pathological Characteristics and Treatment Details

	All RP n=3175	All EBRT n=1830	All EBRT+BT n=999	Optimal RP n=1600	Optimal EBRT n=879	Optimal EBRT+BT n=461
Radical Prostatectomy Patients						
Pathological T stage						
2	786 (25%)	n/a	n/a	459 (29%)	n/a	n/a
3a	980 (31%)			554 (35%)		
3b	1331 (42%)			561 (35%)		
4	65 (2%)			25 (2%)		
Treatment Effect	13 (0%)			1 (0%)		
Pathological Gleason Grade Group						
1	46 (1%)	n/a	n/a	33 (2%)	n/a	n/a
2	629 (20%)			466 (29%)		
3	981 (31%)			578 (36%)		
4	312 (10%)			178 (11%)		
5	1170 (37%)			344 (22%)		
Treatment Effect	38 (1%)			1 (0%)		
Positive margins	1370 (43%)	n/a	n/a	654 (37%)	n/a	n/a
Positive lymph nodes	1174 (37%)	n/a	n/a	357 (20%)	n/a	n/a
RP alone	1587 (50%)			744 (47%)		
Neoadjuvant systemic therapy alone	275 (9%)			77 (5%)		
Adjuvant radiotherapy	347 (11%)			198 (12%)		
Adjuvant systemic therapy alone	120 (4%)			70 (4%)		
Salvage radiotherapy	847 (27%)			511 (32%)		
Radiotherapy Patients						
Upfront androgen deprivation therapy usage		1395 (76%)	897 (90%)		879 (100%)	420 (100%)
Duration of androgen deprivation therapy, median (IQR) (months)		22 (12-30)	12 (4-24)		28 (24-36)	24 (18-24)
Brachytherapy type						
Low dose rate			353 (35%)			72 (16%)
High dose rate			646 (65%)			389 (84%)
All Patients						
Local salvage	847 (27%)	18 (1%)	4 (0%)	511 (34%)	8 (1%)	0 (0%)
Systemic salvage	468 (15%)	319 (17%)	93 (9%)	215 (14%)	150 (17%)	15 (3%)

EBRT, external beam radiotherapy; EBRT+BT, external beam radiotherapy with brachytherapy boost; n, number; RP, radical prostatectomy

© 2021 Kishan AU et al. *JAMA Network Open.*

eTable 3. Specific Breakdown of Presence of Adverse Clinicopathologic Features by Cohort

Characteristic	All RP n=3175	All EBRT n=1830	All EBRT+BT n=999	Optimal RP n=1600	Optimal EBRT n=879	Optimal EBRT+BT n=461
Primary Gleason Pattern 5	600 (19%)	166 (9.1%)	165 (18.2%)	123 (7.7%)	78 (8.9%)	60 (15.7%)
50% percent cores positive	2398 (89.5%)	1467 (89%)	558 (80.9%)	1276 (91.6%)	707 (86.4%)	219 (85.5%)
cT3b-4	154 (5%)	308 (17%)	135 (13.6%)	84 (5.4%)	176 (20.3%)	62 (13.5%)
≥2 NCCN High risk features	2264 (71.3%)	1035 (56.6%)	459 (45.9%)	1191 (74.4%)	458 (52.1%)	178 (38.6%)
Multiple unfavorable features in one patients	338 (10.6%)	281 (15.4%)	146 (14.6%)	82 (5.1%)	159 (18.1%)	61 (13.2%)
Unfavorable by						
≥2 NCCN High risk features only	368 (11.6%)	189 (10.3%)	304 (30.4%)	200 (12.5%)	90 (10.2%)	191 (41.4%)
Other features only	2264 (71.3%)	1035 (56.6%)	459 (45.9%)	1191 (74.4%)	458 (52.1%)	178 (38.6%)
Both	543 (17.1%)	606 (33.1%)	236 (23.6%)	209 (13.1%)	331 (37.7%)	92 (20%)

EBRT, external beam radiotherapy; EBRT+BT, external beam radiotherapy with brachytherapy boost; n, number; NCCN, National Comprehensive Cancer Network; RP, radical prostatectomy

The percentages in any individual characteristics may add up to >100%, as any given patient may have more than 1 unfavorable risk characteristic.

© 2021 Kishan AU et al. *JAMA Network Open.*

eTable 4. Covariate Effect Sizes Before and After Inverse Probability of Treatment Weighting Across Treatment Groups

Parameter	All patients		Appropriate Multimodality Treatment	
	Unweighted Effect size	Weighted Effect Size	Unweighted Effect size	Weighted Effect Size
Age	0.23	0.16	0.23	0.14
Initial PSA	0.15	0.08	0.18	0.14
Biopsy Gleason Grade Group	0.12	0.05	0.08	0.05
Clinical Tumor Stage	0.30	0.29	0.37	0.35

Effect sizes were determined using the partial eta-squared method before and after inverse probability of treatment weighting. Inverse probability of treatment weights were calculated using propensity scores that were determined using multinomial logistic regression with treatment cohort as the outcome and the presence of unfavorable disease (yes/no) and site-centered age at treatment, ln(initial PSA), clinical T stage, Gleason Grade group as pre-treatment prognostic covariates.

© 2021 Kishan AU et al. *JAMA Network Open.*

eTable 5. Clinical Characteristics After Propensity Score Adjustment

	All RP (3261.8)	All EBRT (1678.3)	All EBRT + BT (955.5)	p-value	Optimal RP (1781.3)	Optimal EBRT (750.9)	Optimal EBRT + BT (350.7)	p-value
Median Follow-up (IQR)	3.82 (2.07-6.21)	6.78 (3.99-10.21)	7.22 (4.23-9.92)	<0.001	3.99 (2.09-6.41)	7.28 (4.49-10.61)	8.02 (5.87-10.56)	<0.001
Age, median (IQR) (years)	65.3 (59.6-70)	67 (60-73)	68 (62.2-74)	<0.001	65.4 (60-70)	68 (61.9-74)	67 (62-72.1)	<0.001
Initial PSA, median (IQR) (ng/mL)	13.4 (6.3-28)	14 (6.8-28.7)	17.4 (8-29.8)	0.005	12 (6.4-27.4)	14.1 (6.8-31.2)	21.4 (10.3-34)	0.6
Biopsy Gleason Grade Group				<0.001				<0.001
1	214.6 (6.6%)	47.5 (2.8%)	65.2 (6.8%)		98.5 (5.5%)	26.2 (3.5%)	36.9 (10.5%)	
2	351.4 (10.8%)	168.6 (10.1%)	76.2 (8%)		180.6 (10.1%)	80.3 (10.7%)	35.2 (10%)	
3	353 (10.8%)	146.7 (8.7%)	139.9 (14.6%)		190.6 (10.7%)	66.6 (8.9%)	75.2 (21.4%)	
4	1414 (43.4%)	747.1 (44.5%)	338.2 (35.4%)		1155.3 (64.9%)	324.7 (43.3%)	103.1 (29.4%)	
5	928.7 (28.5%)	568.4 (33.9%)	335.9 (35.2%)		156.4 (8.8%)	253 (33.7%)	100.3 (28.6%)	
Clinical Tumor Category (%)				<0.001				<0.001
1c	1488.8 (45.6%)	489.2 (29.2%)	197.1 (20.6%)		843.5 (47.4%)	204.5 (27.2%)	60.6 (17.3%)	
2a	580.4 (17.8%)	274.1 (16.3%)	82.3 (8.6%)		347.9 (19.5%)	122.8 (16.4%)	24.7 (7%)	
2b	443.7 (13.6%)	230.9 (13.8%)	134.1 (14%)		222.3 (12.5%)	108.4 (14.4%)	27.7 (7.9%)	
2c	249.6 (7.7%)	152.4 (9.1%)	110.7 (11.6%)		102.4 (5.8%)	65.1 (8.7%)	48.6 (13.9%)	
3a	339.1 (10.4%)	275 (16.4%)	285.2 (29.9%)		180.9 (10.2%)	134.7 (17.9%)	132.1 (37.7%)	
3b	143.7 (4.4%)	199.8 (11.9%)	125.3 (13.1%)		75.6 (4.2%)	93.8 (12.5%)	51.4 (14.7%)	
4	16.5 (0.5%)	56.8 (3.4%)	20.9 (2.2%)		8.8 (0.5%)	21.5 (2.9%)	5.6 (1.6%)	

EBRT, external beam radiotherapy; EBRT+BT, external beam radiotherapy with brachytherapy boost; IQR, interquartile range; RP, radical prostatectomy; Continuous variables across treatments are compared using Kruskal-Wallis test. The association between treatment and categorical variables are assessed using chi-square test of association

© 2021 Kishan AU et al. *JAMA Network Open.*

eTable 6. Cause-Specific Regression Models of Time Until Prostate Cancer-Specific Mortality and Distant Metastasis

	Hazard Ratio (95%CI)	p-value
**<u>All Patients with Unfavorable Disease</u>**		
**Prostate Cancer Specific Mortality**		
All EBRT *versus* All RP	**0.73 (0.59-0.91)**	**0.006**
All EBRT+BT *versus* All RP	**0.65 (0.49-0.86)**	**0.003**
All EBRT+BT *versus* All EBRT	0.89 (0.67-1.18)	0.418
**Distant Metastasis**		
All EBRT *versus* All RP	**0.5 (0.44-0.58)**	**<0.001**
All EBRT+BT *versus* All RP	**0.29 (0.23-0.36)**	**<0.001**
All EBRT+BT *versus* All EBRT	**0.58 (0.46-0.73)**	**<0.001**
**<u>Optimally Treated Men with Unfavorable Disease</u>**		
**Prostate Cancer Specific Mortality**		
Optimal EBRT *versus* Optimal RP	0.69 (0.47-1.01)	0.054
Optimal EBRT+BT *versus* Optimal RP	0.77 (0.47-1.25)	0.288
Optimal EBRT+BT *versus* Optimal EBRT	1.12 (0.68-1.83)	0.653
**Distant Metastasis**		
Optimal EBRT *versus* Optimal RP	**0.48 (0.38-0.61)**	**<0.001**
Optimal EBRT+BT *versus* Optimal RP	**0.25 (0.17-0.38)**	**<0.001**
Optimal EBRT+BT *versus* Optimal EBRT	**0.52 (0.34-0.81)**	**0.003**

CI, confidence interval; EBRT, external beam radiotherapy; EBRT+BT, external beam radiotherapy with brachytherapy boost; RP, radical prostatectomy

Models are adjusted for sites as random effect with inverse probability of treatment weights, calculated using propensity scores that were determined using multinomial logistic regression with treatment cohort as the outcome and the presence of unfavorable disease (yes/no) and site-centered age at treatment, ln(initial PSA), clinical T stage, Gleason Grade group as pre-treatment prognostic covariates. These models are doubly robust (i.e., were also adjusted for each of those covariates including the propensity score calculation).

© 2021 Kishan AU et al. *JAMA Network Open.*

eTable 7. Cause-Specific Regression Models of Time Until All-Cause Mortality

	**Hazard Ratio & 95%CI**	**p-value**
**<u>All Patients with Unfavorable Disease</u>**		
**All EBRT versus All RP**	**0.77 (0.68-0.87)**	**<0.001**
**All EBRT+BT versus All RP**	**0.84 (0.73-0.97)**	**0.016**
All EBRT+BT versus All EBRT	1.1 (0.95-1.27)	0.225
**<u>Optimally Treated Men with Unfavorable Disease</u>**		
**Optimal EBRT versus Optimal RP**	**0.56 (0.46-0.68)**	**<0.001**
Optimal EBRT+BT versus Optimal RP	**0.75 (0.6-0.94)**	**0.012**
Optimal EBRT+BT versus Optimal EBRT	**1.34 (1.05-1.69)**	**0.016**

CI, confidence interval. EBRT, external beam radiotherapy; EBRT+BT, external beam radiotherapy with brachytherapy boost; RP, radical prostatectomy

Models are adjusted for sites as random effect with inverse probability of treatment weights, calculated using propensity scores that were determined using multinomial logistic regression with treatment cohort as the outcome and the presence of unfavorable disease (yes/no) and site-centered age at treatment, ln(initial PSA), clinical T stage, Gleason Grade group as pre-treatment prognostic covariates. These models are doubly robust (i.e., were also adjusted for each of those covariates including the propensity score calculation). Note that proportional hazards were not met.

© 2021 Kishan AU et al. *JAMA Network Open.*
